# Some sage advice: A case report of sage burning causing interstitial lung disease

**DOI:** 10.1016/j.clinme.2025.100337

**Published:** 2025-06-08

**Authors:** Jonathan Ayling-Smith, Richard Attanoos, Nicola-Xan Hutchinson

**Affiliations:** a Department of Respiratory Medicine, Cardiff and Vale University Health Board, Cardiff, Wales, UK; b Department of Cellular Pathology, University Hospital of Wales, Cardiff, Wales, UK; c School of Medicine, Cardiff University, Cardiff, UK

**Keywords:** Case report, Sage, Burning, ILD, RB-ILD, Biomass

## Abstract

**Background:**

Cigarette smoking remains the commonest cause of chronic obstructive pulmonary disease (COPD) and respiratory bronchiolitis-associated interstitial lung disease (RB-ILD). Biomass combustion remains a high risk for causing respiratory disease.

**Method:**

We present a case of a 41-year-old woman and never-smoker with worsening breathlessness. It was identified that she made fire pits burning sage and oils for spiritual work, but beyond this had no respiratory risk factors.

**Results:**

Radiological evidence of RB-ILD was identified and a bronchoscopic lavage demonstrated the presence of striking green pigmented macrophages, presumed to be chlorophyll uptake.

**Conclusions:**

Cessation of burning sage exposure has resulted in a positive outcome. This case highlights the need for an extensive occupational or exposure history in respiratory medicine and describes histological features of interstitial lung disease not previously identified.

## Highlights

• A detailed employment history is vital in identifying less common exposures or to challenge the clinician’s preconceptions about what an occupation involves.
• Smoke inhalation may come from other sources beyond first-hand tobacco smoke and may not be identified without targeted questioning.
• The nature of the components of a fire such as the accelerant, fuel, perfumes and containment may help establish the significance of the exposure.
• Here, significant repeated exposure to burning organic matter has led to interstitial lung disease, which is evidenced clinically, radiologically and pathologically in a way not previously described.

## Case presentation

A 41-year-old woman of Afro-Caribbean ethnicity presented to the acute medical take with a 4-month history of progressive shortness of breath and lethargy. This breathlessness was affecting her activities of daily living and was associated with a non-productive cough. There were no associated cardiovascular symptoms.

She was a lifelong non-smoker, but had an extensive passive smoking history within her family and during her previous job as a substance misuse worker. At time of presentation, she worked in a funeral home and also did spiritual work including making fire pits using incense, and burning oils and sage. She had no exposure to birds, feathers, damp or mould. There was no evidence of *Mycobacterium tuberculosis* (TB) exposure or family history of lung disease.

She had a background history of antibody-positive limbic encephalitis (not currently requiring treatment), migraines, anxiety and vitamin B12 deficiency. Her drug history included sterexol D3, pregabalin, amitriptyline, fluoxetine and sumatriptan.

On clinical examination, there was small cervical lymphadenopathy. There was mild wheeze in the right upper zone on auscultation of the chest. There was mild leg bruising but no erythema nodosum.

**Fig. 1:**
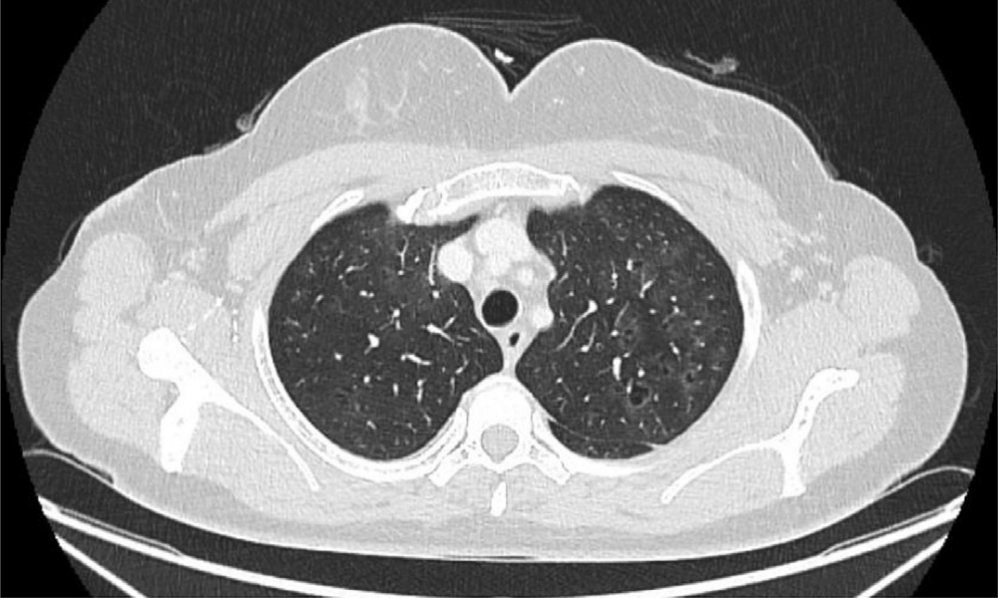
Apical slice of CT thorax demonstrating subtle peribronchovascular groundglass opacification predominantly within the upper lobes and mild upper lobe predominant centrilobular emphysema.

Initial blood tests for HIV, avian precipitins, *Aspergillus* serology, serum angiotensin-converting enzyme (ACE), anti-neutrophil cytoplasmic antibody (ANCA), anti-nuclear antibodies (ANA) and rheumatoid factor were negative. Her full blood count was unremarkable and her serum C-reactive protein (CRP) was 1 mg/L. Her total immunoglobulin E (IgE) level was 136 kU/L. An electrocardiogram (ECG) and initial chest radiograph were reported to be normal. Following this, a computed tomography (CT) thorax, abdomen and pelvis demonstrated sub-centimetre, reactive mediastinal lymph nodes, as well as subtle peribronchovascular groundglass opacification predominantly within the upper lobes and mild upper lobe predominant centrilobular emphysema (Fig. 1). Lung function tests demonstrated an forced expiratory volume in 1 s (FEV1) 3.05 L (102%), forced vital capacity (FVC) 4.34 (119%), ratio 68%, transfer factor for carbon monoxide (TLCO) 73% and carbon monoxide transfer coefficient (KCO) 69%.

The differential diagnoses here included sarcoidosis, inhalational injury, hypersensitivity pneumonitis and smoking-related interstitial lung disease (through passive smoking).

**Fig. 2:**
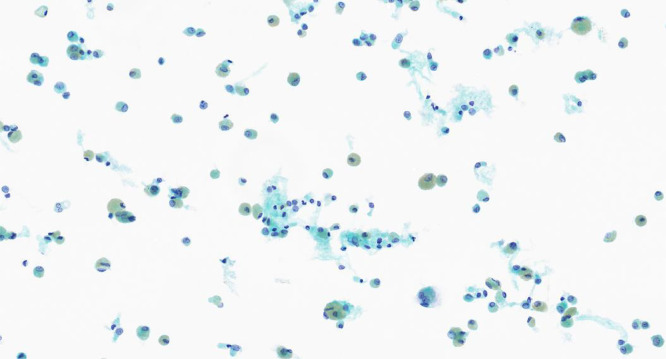
BAL sample stained with Papanicolaou stain 40x magnification shows prominent numbers of alveolar macrophages, many of which have prominent green pigment consistent with chlorophyll from combustion of organic common sage (*Salvia officinalis* plant).

In order to differentiate these, a bronchoscopy with bronchoalveolar lavage was organised. The macroscopic appearances were unremarkable. Targeted upper lobe washings grew *Haemophilus influenzae* and *Staphylococcus aureus,* which was subsequently treated. The bronchoalveolar lavage (BAL) demonstrated 90% macrophages which were pigmented green (Fig. 2) and lymphocytes 10%. No malignant cells were seen.

This patient’s case was discussed in the local interstitial lung disease multidisciplinary team (MDT) and a diagnosis of respiratory bronchiolitis-associated interstitial lung disease (RB-ILD) was made with associated inhalation injury in keeping with the burning of organic matter, likely the sage. Cessation of her burning practices was recommended.

The patient was reviewed in outpatients 6 months later and was found to have an improvement in her respiratory symptoms since stopping these practices. She was subsequently discharged and has had no further contact with healthcare services regarding this problem.

## Discussion

Fire pits and open fire cooking remain the leading cause of COPD globally. However, smoking is a more common cause in the UK.^1^^,^^2^ As medical professionals, the practice of taking a smoking history is routine, but further interrogation about other sources of smoke inhalation is less practised.

Similarly, the importance of an employment history is well understood. However, it is increasingly less common for a population to remain in the same vocation lifelong and so more detail will be required in order to identify individuals at risk of occupational disease. In this particular case, the diagnosis of sage inhalation injury through spiritual practices was only elicited through a discussion about the patient’s social history and the nature of her employment, which would not have been elucidated through the usual simple employment screening questions.

A key pathological feature of respiratory bronchiolitis is the presence at lavage of brown-coloured macrophages, which can also be found in the airways of otherwise healthy current smokers.^3^ There were no reports found in the literature of green-pigmented macrophages on bronchoalveolar lavage. It is likely that this is a reflection of macrophage phagocytosis of chlorophyll molecules from the incomplete combustion of sage. Smoking cessation is the mainstay of management of RB-ILD, alongside supportive measures such as pulmonary rehabilitation, vaccination and supplemental oxygen. The use of steroids is controversial as the evidence is conflicting.^3^

## Funding

This research did not receive any specific grant from funding agencies in the public, commercial or not-for-profit sectors.

## Consent for publication

Written consent has been obtained from the patient described in the case report for the reporting of this condition, including the anonymised figures.

## CRediT authorship contribution statement

**Jonathan Ayling-Smith:** Writing – original draft, Project administration, Conceptualization. **Richard Attanoos:** Writing – review & editing, Visualization, Data curation. **Nicola-Xan Hutchinson:** Writing – review & editing, Supervision, Conceptualization.

## Declaration of competing interests

The authors declare that they have no known competing financial interests or personal relationships that could have appeared to influence the work reported in this paper.
